# Monitoring the Early Response of Fulvestrant Plus Tanshinone IIA Combination Therapy to Estrogen Receptor-Positive Breast Cancer by Longitudinal ^18^F-FES PET/CT

**DOI:** 10.1155/2019/2374565

**Published:** 2019-06-10

**Authors:** SiMin He, MingWei Wang, YongPing Zhang, JianMin Luo, YingJian Zhang

**Affiliations:** ^1^Department of Nuclear Medicine, Fudan University Shanghai Cancer Center, Shanghai, China; ^2^Department of Oncology, Shanghai Medical College, Fudan University, Shanghai, China; ^3^Center for Biomedical Imaging, Fudan University, Shanghai, China; ^4^Shanghai Engineering Research Center of Molecular Imaging Probes, Shanghai, China

## Abstract

Endocrine monotherapy of breast cancers is generally hampered by the primary/acquired resistance and adverse sides in clinical settings. Herein, advantaging the multitargeting antitumor effects and normal organ-protecting roles of Chinese herbal medicine, the aim of this study was to investigate the enhanced synergistic efficacy of fulvestrant plus Tan IIA combination therapy in ER-positive breast cancers and to monitor the early response by longitudinal ^18^F-FES PET/CT imaging. The experimental results showed FUL + Tan IIA combination therapy significantly inhibited tumor growth of ER-positive ZR-75-1 tumor xenografts and exhibited distinct antitumor effects at an earlier time point after treatment than did the monotherapy of FUL or Tan IIA. Moreover, ^18^F-FES PET/CT imaging competently monitored the early response of FUL + Tan IIA combination therapy. The quantitative ^18^F-FES %ID/g_max_ in vivo was further confirmed by and correlated well with ER*α* expression ex vivo. In conclusion, the synergic effect of FUL + Tan IIA combination therapy to ER-positive breast cancers was verified in the preclinical tumor models and the early treatment response could be monitored by ^18^F-FES PET/CT.

## 1. Introduction

Endocrine therapy is one of the major treatments of breast cancer and continues to be the cornerstone of treatment for estrogen receptor-positive (ER^+^) breast cancer due to its effectiveness and favorable toxicity profile [1]. However, clinical dilemmas about endocrine therapy still remain due to intrinsic or acquired resistance. For example, the objective response rate to second-line endocrine therapy is less than 20% and even first-line endocrine therapy also only 30–50% [2, 3]. Thus, it is important to develop the combination therapy of endocrine drugs plus other medicines as new treatment strategies, targeting multiple signaling pathways, to improve the therapeutic outcome of ER-positive breast cancers by applying a double-striking effect to amplify antitumor potentials and/or overcome resistance to endocrine treatment.

Traditional Chinese medicine (TCM), based on Chinese herbal medicines, holds a very popular, unique, and lone-term practical experience in China and most regions of the world. It has been reported that >90% of modern Chinese cancer patients have received some form of TCM during their treatment regimen [4]. Increasing evidences suggest that a variety of bioactive compounds or phytochemicals from Chinese herbal medicines can be combined with other antitumor drugs to enhance the total efficacy and also to help reduce the adverse effects [5, 6]. Tanshinone IIA (Tan IIA, 14, 16-epoxy-20-nor-5 (10), 6, 8, 13, 15-abietapentaene-11, 12-dione), isolated from the Chinese medicinal herb Danshen (root of Salvia miltiorrhiza BUNGE), has been successfully used in clinics for the treatment of coronary heart diseases, angina, myocardial infarction, and cerebrovascular diseases with minimal side effects [7, 8]. Recently, Tan IIA as a multitarget drug has attracted great attentions in cancer therapy due to its potential anticancer activities [9]. For instance, Tan IIA inhibited the growth and induced the apoptosis of breast cancer cells through multiple mechanisms, including activation of caspase-3, increasing the Bax to Bcl-xL ratios, as well as epigenetic modification of Aurora-A expression [10–12]. In addition, Tan IIA inhibited angiogenesis and tumor growth through repression of HIF-1*α* at the translation level in human breast cancer xenografts [13, 14]. Recent evidence suggests that Tan IIA significantly inhibited PI3K/Akt/mTOR signaling [15]. Moreover, enhanced synergistic effects of Tan IIA in combination therapy with anticancer chemotherapeutics have been observed and have obtained increasing research efforts against human cancers [16]. However, rare literature studies are available to investigate the enhanced efficacy of Tan IIA plus endocrine drug combination therapy in ER-positive breast cancers.

Along with the development of new anti-cancer strategies, it is urgently needed for the advanced medical imaging methods to early assess treatment effect and timely discriminate between responders and nonresponders. The conventional method for monitoring treatment responses relies on changes of tumor size based on anatomical imaging like CT and MR according to response evaluation criteria in solid tumors (RECIST) [17, 18]. However, the time until tumor size shrinkage can be as long as many weeks, even months after therapy initiation. As a result, the repeated tumor volume measurements monthly or weekly for RECIST are often required. Noninvasive molecular imaging, such as positron emission tomography (PET), allows detecting early biological response after anticancer treatment, thus would be valuable to distinguish responders from nonresponders before changes in tumor volume become evident [19]. ^18^F-fluorodeoxyglucose (^18^F-FDG), reflecting the glucose metabolism of cancer cells, is the most widely used PET tracer for the evaluation of therapeutic efficacy [20, 21]. However, current studies reported that ^18^F-FDG PET only played a limited role in the early response assessment and prediction of endocrine therapy of breast cancer [22]. 16*α*-[^18^F]-fluoro-17*β*-estradiol (^18^F-FES) is an ER-specific imaging probe, and ^18^F-FES PET has been extensively performed for the noninvasive imaging and measurement in vivo of ER expression breast cancer because ^18^F-FES uptake correlates well with the immunohistochemical scoring for ER [23–25]. The comparison study of ^18^F-FES and ^18^F-FDG showed that ^18^F-FES PET/CT imaging could predict and monitor the response to endocrine therapy in ER^+^ breast cancer early. Moreover, ^18^F-FES PET/CT also stepped into the application of early response prediction to neoadjuvant chemotherapy of breast cancer [26].

Herein, the aim of this study was to investigate the enhanced synergistic efficacy of fulvestrant plus Tan IIA combination therapy in ER-positive breast cancers and to monitor the early response by longitudinal ^18^F-FES PET/CT imaging.

## 2. Materials and Methods

### 2.1. Cell Culture

ZR-75-1 cell lines were obtained from Cell Bank, Shanghai Institutes for Biological Sciences, Chinese Academy of Sciences. The cells were grown in RPMI 1640 medium with L-glutamine, penicillin 100 *μ*g/mL, streptomycin 100 *μ*g/mL, and 10% fetal calf serum in a humidified 5% CO_2_ atmosphere at 37°C. It should be mentioned that we followed the methods of He et al. for cell culture, tumor model, microPET/CT imaging, and ER*α* scoring, which was already developed by us [22].

### 2.2. ER^+^ Breast Cancer Model Xenografts

The animal experiment was approved by the Institutional Animal Care and Use Committee of Fudan University (LASFDI-20140179A032). All procedures involving animals were performed in accordance with institutional guidelines. 72 female Balb/c nude mice (5 weeks) were obtained from the Department of Laboratory Animal Science, Fudan University, and allowed to acclimatize for one week in the animal facility before any intervention was initiated. Estrogen pellets (0.72 mg, 90 day release, Innovative Research of America) were implanted into the mouse body three days prior to tumor cell inoculation and remained until the tumors reached 6.0–8.0 mm (about 14 days after inoculation). The mice were injected subcutaneously with ER^+^ human breast cancer ZR-75-1 cells (5 × 10^6^ cells in 100 *μ*L medium mixed with 100 *μ*L Matrigel chambers (BD Biosciences)) into the mammary fat pad on the right thorax. All invasive procedures to the animals were done under anesthesia (3% pentobarbital sodium 40 mg/kg). The tumor growth was followed up by the caliper (model 530–312; range 0–150 mm; Mitutoyo, Kawasaki, Kanagawa, Japan) measurements of perpendicular axes. The tumor volume was calculated by the following formula: *V*_*x*_ (*V*_*x*_ = *ab* ^2^/2), where *V*_*x*_ is the tumor volume on the day *x* after treatment, *a* is the long diameter of the xenograft, and *b* is the short diameter of the xenograft [27].

### 2.3. Therapy Protocols

**Figure 1:**
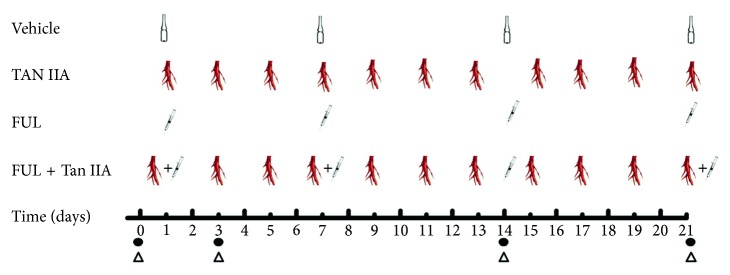
Experimental design for treatment protocols, ^18^F-FES microPET/CT imaging, and immunohistochemistry staining. Vehicle (0.9% sodium chloride, 50 *μ*l/mouse/week, s.c.), Tan IIA (30 mg/kg, every other day, injected via tail vein), FUL (250 mg/kg, weekly, s.c.), and FUL + Tan IIA combination therapy. The entire period of treatment was 21 days. •: ^18^F-FES microPET/CT imaging; ^△^IHC staining of ER*α*.

Fulvestrant (FUL, 0.25 g/5 ml, FASLODEX®, AstraZeneca) and sulfotanshinone IIA sodium (Tan IIA, 5 mg/ml, from the first Biochemical Pharmaceutical Co., Ltd, Shanghai, China) were used for the therapies of ER^+^ breast cancer models xenografts. When the tumors reached a measurable size (6.0–8.0 mm), mice were randomly assigned to four groups (*n*=18) to treat, and the pellets were surgically removed before the initiation of treatments. The dose and protocol of each group was as the followings (Figure 1): vehicle (0.9% sodium chloride, 50 *μ*l/mouse/week, s.c.), Tan IIA (30 mg/kg, every other day, injected via tail vein), FUL (250 mg/kg, weekly, s.c.), FUL + Tan IIA combination therapy, which were made according to the published literatures [10, 28]. The entire period of treatments was 21 days.

### 2.4. MicroPET/CT Imaging and Quantitative Analysis

^18^F-FES was produced using a modified Explora FDG_4_ module (Siemens) in our center as previously reported [29]. MicroPET/CT (Inveon, Siemens) scanning was performed on days 0, 3, 14, and 21 after treatment with injection of 5.55 MBq (150 *μ*Ci) of ^18^F-FES into the tail vein. 10 min static PET scans and CT imaging were acquired at 60 min after the injection of ^18^F-FES. Isoflurane was administered 10 minutes before the scanning, and mice were maintained under anesthesia during the scanning period. The images were reconstructed using three-dimensional ordered-subset expectation maximization (OSEM3D)/maximum algorithm. For data analysis, the region of interest (ROI) was manually drawn to cover the whole tumor on fused PET/CT images. A similar ROI was drawn on the muscle of the opposite foreleg. The max of percentage-injected dose per Gram (%ID/g_max_) and standardized uptake values (SUV_max_) of the tumor and muscle in the ROIs were recorded. %ID/g before and after therapy was denoted as %ID/g day_0_ and %ID/g day_*n*_, respectively. Changes after therapy are denoted as Δ%ID/g = (%ID/g day_*n*_ − %ID/g day_0_)/%ID/g day_0_ × 100%.

### 2.5. Immunohistochemistry

Three mice in each group were sacrificed to collect tumor tissue for IHC analysis at the corresponding imaging time points. An ER*α* (Santa Cruz Biotechnology, TX, USA) antibody was used at 1 : 35 dilution, with a 10-min high temperature antigen retrieval in citrate buffer (pH = 6.0). Immunoreactivity was detected by using the EnVision + System (DAKO) with diaminobenzidine chromogen according to the manufacturer's protocol. Known positive and negative controls (obtained by omission of primary antibodies) were used as a quality control of the staining. For ER*α* evaluation, the total proportion of cells staining positively at any intensity was scored into five levels: 0 (no cells staining), 1 (1–25% cells stained), 2 (26–50% cells stained), 3 (50–75% cells stained) or 4 (>75% cells stained).

### 2.6. Statistical Analysis

Data were expressed as the mean ± SD. Statistical analyses were performed using variance (ANOVA) models. Pearson correlation coefficients were calculated to determine the correlation of ^18^F-FES uptake with ER*α* expression. *P* values < 0.05 were considered statistically significant. All statistical analyses were performed using the SPSS 19.0 software package (SPSS-IBM).

## 3. Results

### 3.1. FUL + Tan IIA Combination Therapy Significantly Inhibited Tumor Growth of ER^+^ Breast Cancer ZR-75-1 Xenografts

**Figure 2:**
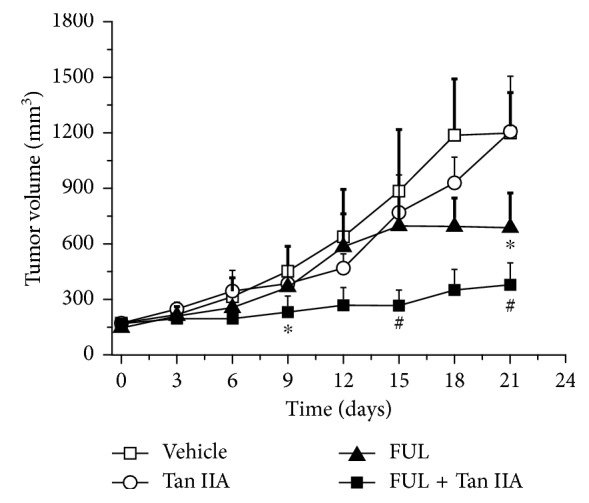
Effect of FUL + Tan IIA combination therapy and monotherapy of FUL, Tan IIA, and vehicle on the growth of ER-positive ZR-75-1 tumor xenografts. ^*∗*^ *P* < 0.05, ^#^ *P* < 0.001 compared to the vehicle group.

As shown in Figure 2, FUL + Tan IIA combination therapy exhibited the distinct anti-tumor effect, compared with monotherapy of FUL or Tan IIA. As early as on day 9 after treatment, FUL + Tan IIA combination therapy led to a significant delay of tumor growth, resulting into a significant difference of tumor size (231 ± 87 mm^3^ vs 451 ± 137 mm^3^, *P* < 0.05), compared with the Vehicle group. However, monotherapy of FUL or Tan IIA alone did not significantly delay tumor growth, that is, no significant difference of the tumor size on day 9 (382 ± 102 vs 451 ± 137, 365 ± 25 vs 451 ± 137, *P* > 0.05), compared with the Vehicle group. As a contrast, there was a significant difference of tumor size between FUL group and the Vehicle group (688 ± 188 mm^3^ vs 1194 ± 224 mm^3^, *P* < 0.05) until on day 21 after treatment. On the contrary, there was no significant difference of the tumor size between Tan IIA group and the Vehicle group at any time point (*P* > 0.05), yet with slight inhibition of tumor growth. These results implicated that FUL + Tan IIA combination therapy could be an effective therapeutics for ER-positive breast cancers own to the enhanced anti-tumor potentials.

### 3.2. ^18^F-FES microPET/CT Imaging Monitored the Early Response of FUL + Tan IIA Combination Therapy to ER-Positive Breast Cancer ZR-75-1 Xenografts

**Figure 3:**
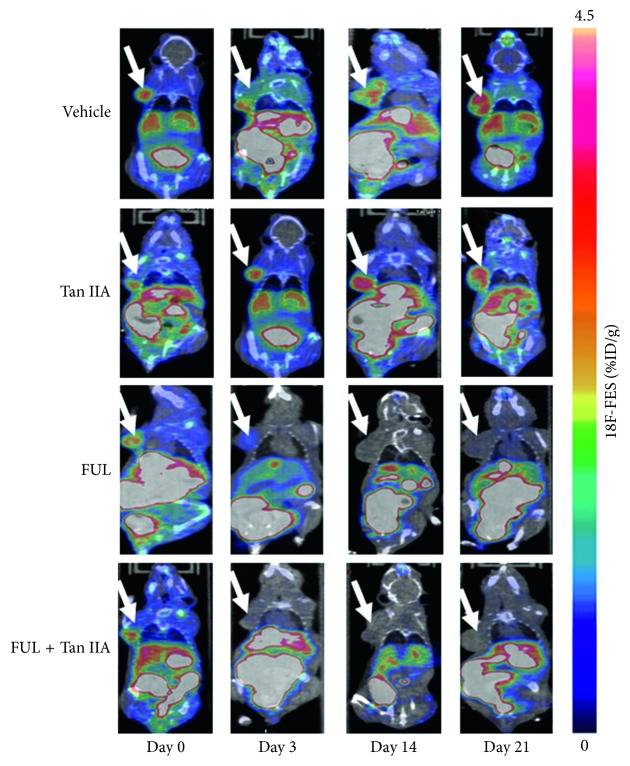
^18^F-FES microPET/CT imaging of ER-positive ZR-75-1 tumor mice on days 0, 3, 14, and 21 after treatment. The tumors were indicated by arrows.

**Figure 4:**
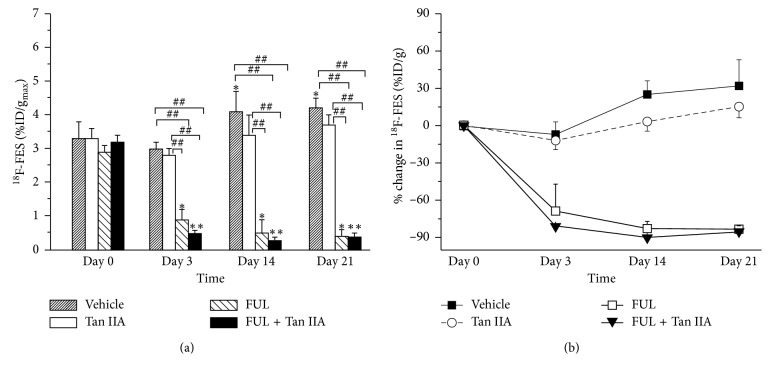
Quantitative analysis of ^18^F-FES uptake %ID/g_max_ (a) and the change Δ%ID/g_max_ (b) from the microPET/CT imaging on days 0, 3, 14, and 21 after treatment. ^*∗*^ *P* < 0.05, ^*∗∗*^ *P* < 0.001, within groups compared to baseline; ^#^ *P* < 0.05, ^##^ *P* < 0.001 between treatment and vehicle groups.

The longitudinal ^18^F-FES microPET/CT images of ZR-75-1 tumor mice were shown in Figure 3, and the quantitative uptake of ^18^F-FES in tumor lesions at each time point in Figure 4. As shown in Figure 4, there was no difference in ^18^F-FES %ID/g_max_ at baseline between all groups (*P* > 0.05). The tumor uptake of ^18^F-FES in FUL + Tan IIA group decreased remarkably from 3.2 ± 0.2 at baseline to 0.5 ± 0.09 (−81 ± 2%, *P* < 0.001) on day 3, an early time point, to 0.3 ± 0.08 (−90 ± 2%, *P* < 0.001) on day 14, to 0.4 ± 0.1 (−85 ± 4%, *P* < 0.001) on day 21 after treatment initiation. Notably, the change of ^18^F-FES uptake in FUL + Tan IIA group shared an identical decrease trend with that in FUL group. Moreover, there was no statistical difference for ^18^F-FES uptake in ZR-75-1 tumor between FUL + Tan IIA group and FUL group at each time point. As contrast, ^18^F-FES uptake in tumor lesions in both Tan IIA group and the vehicle group slightly increased over time with identical increase trend, and no significant difference was found within and between these two groups at any time point (*P* > 0.05). Moreover, ^18^F-FES uptakes were also analyzed by the quantitative SUV_max_ (Figure S1), and it was found that SUV_max_ data shared the identical treads with %ID/g_max_ data, which further confirmed the findings above.

### 3.3. FUL + Tan IIA Combination Therapy Significantly Reduced ER Expression in ZR-75-1 Tumor Lesions

**Figure 5:**
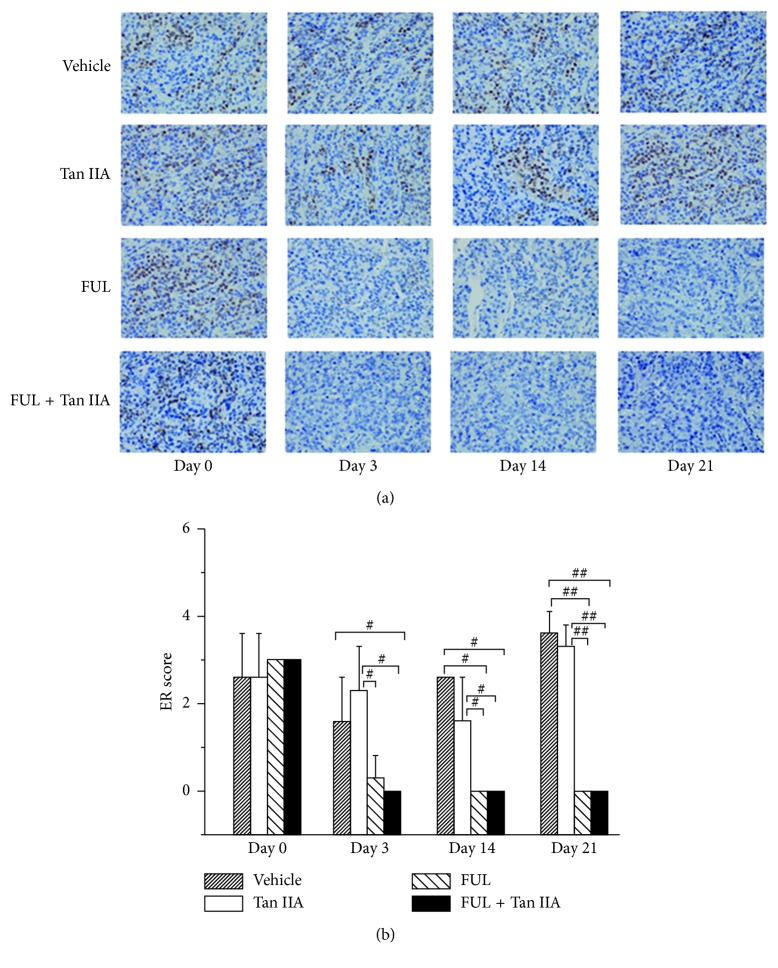
IHC staining and semiquantitative scores of ER*α* expression at the corresponding imaging time points. ^#^ *P* < 0.05, ^##^ *P* < 0.001 between groups.

The IHC staining ex vivo and qualitative scores of ER expression in ZR-75-1 tumor lesions were presented in Figure 5. According to Figure 5, no difference was found in ER expression at baseline between all groups (*P* > 0.05). The ER expression in ZR-75-1 tumor lesions in FUL + Tan IIA group decreased dramatically from 3 at baseline to 0 (*P* < 0.001) on day 3, an early time point, then kept lowest status until day 14 and day 21 after treatment initiation. Importantly, the change of ER expression in FUL + Tan IIA group held a similar decrease trend with that in FUL group. Moreover, there was no significant difference for ER expression in ZR-75-1 tumor between FUL + Tan IIA group and FUL group at each time point. As contrast, ER expression in tumor lesions in both Tan IIA group and the vehicle group remained approximate level over time, and no significant difference was found within and between these two groups at any time point (*P* > 0.05).

### 3.4. ^18^F-FES Uptakes In Vivo Correlated Significantly with IHC Scores Ex Vivo of ER*α* Expression

**Figure 6:**
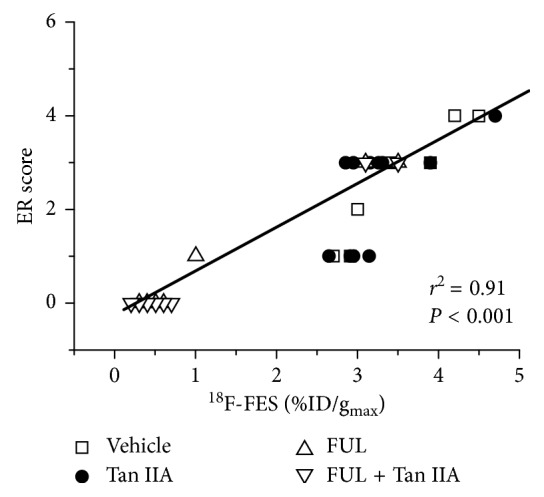
Correlation between %ID/g_max_ of ^18^F-FES uptake and ER*α* IHC scores. There is a significant positive correlation between ^18^F-FES uptake and ER expression (*r* ^2^=0.91, *P* < 0.001).

Figure 6 depicted the correlation of ^18^F-FES %ID/g_max_ in vivo and IHC scores of ER expression ex vivo. Accordingly, it was found that there was a significantly positive correlation between ^18^F-FES uptake and ER*α* expression (*r* ^2^=0.91, *P* < 0.001).

## 4. Discussion

Approximately 75% of breast cancers express the estrogen receptor at diagnosis and are therefore suitable for endocrine therapy, which blocks the mitogenic action of estrogens on breast cancer cells [30]. However, primary or acquired resistance is a major problem limiting the clinical benefit of endocrine monotherapy [31]. Thus, it is of significance to explore new treatment strategies that target multisignaling pathways to enhance the therapy efficacy of ER-positive breast cancers.

Combination therapy of bioactive herbal compounds with modern antitumor drugs opens a new emerging field against human cancers. The combined regimens of Tan IIA with chemotherapeutical drugs have been reported to potentiate the therapy efficacy, to reduce the adverse events, and to improve the in vivo biobehaviors of drugs. In this study, we surveyed the enhanced role of Tan IIA plus FUL combination therapy against ER-positive breast cancers in ZR-75-1 xenograft models. Our results found that Tan IIA plus FUL combination therapy significantly suppressed tumor growth and was more effective than any monotherapy via either FUL or Tan IIA alone (Figure 2). This indicated the synergistically enhanced antitumor effect of Tan IIA plus FUL combination therapy, which was consistent with the literature studies [32].

Following the experimental observations mentioned above, we performed ^18^F-FES microPET/CT as a noninvasive functional molecular imaging in vivo to monitor the early treatment response of Tan IIA plus FUL combination therapy to ER-positive breast cancers. According to the data of microPET/CT imaging, ^18^F-FES %ID/g_max_ of ZR-75-1 tumor lesions in the combination group of Tan IIA plus FUL exhibited the most significant decrease with a mean drop of −81 ± 2% on day 3 after treatment and continued this decrease trend until day 21. It was noted that the change of ^18^F-FES uptake in the FUL group showed a similar decrease trend with the combination group. On the contrary, the %ID/g_max_ values of ^18^F-FES uptake in tumor sites presented a stable increase over time for the Tan IIA group, as well as for the Vehicle group. This implied that Tan IIA alone could not impact the ER*α* expression status and thus ^18^F-FES uptake of breast tumors. Moreover, ^18^F-FES %ID/g_max_ was confirmed by the IHC staining ER*α* expression, both of which correlated well with each other. Therefore, ^18^F-FES PET/CT could discriminate the response of the Tan II plus FUL combination group and the FUL group from the nonresponse of the Tan IIA group and the Vehicle group to ER-positive breast cancers. It should be further clarified that the significant antitumor effect appeared on as early as day 3 for Tan II plus FUL combination, yet as late as day 21 for FUL alone. Consequently, ^18^F-FES PET/CT could monitor the early response of Tan IIA plus FUL combination therapy to ER-positive breast cancers.

There were several limitations in the preliminary study. The first limitation was that only a single ER-positive breast cancer model was involved, which could not represent various types of breast cancers with different ER expressions. Another limitation was that the molecular and biologic mechanisms of Tan IIA plus FUL combination therapy was not performed, which could be important to better understand this new anticancer strategy.

## 5. Conclusion

In summary, longitudinal ^18^F-FES PET/CT was successfully applied in monitoring the early treatment response to fulvestrant plus Tan IIA combination therapy for ER-positive breast cancers. This study could open new potentials of Chinese herbal medicine in combination with endocrine drugs and functional molecular imaging of ^18^F-FES PET/CT for the therapy and efficacy evaluation of ER-positive breast cancers.

## Data Availability

The data used to support the findings of this study are included within the article.

## Supplementary Materials

Supplementary Materials Figure S1: quantitative analysis of ^18^F-FES uptake SUV from the microPET/CT imaging on days 0, 3, 14, and 21 after treatment.
