# Supplementary material for: Monitoring the Early Response of Fulvestrant Plus Tanshinone IIA Combination Therapy to Estrogen Receptor-Positive Breast Cancer by Longitudinal 18F-FES PET/CT
Source: Contrast Media Mol Imaging. 2019 Jun 10;2019:2374565. doi: 10.1155/2019/2374565 (PMC6590558; doi:10.1155/2019/2374565)
Supplement: Supplementary Materials — Figure S1: quantitative analysis of 18F-FES uptake SUV from the microPET/CT imaging on days 0, 3, 14, and 21 after treatment. [file 2374565.f1.docx]

**Supplementary materials:**


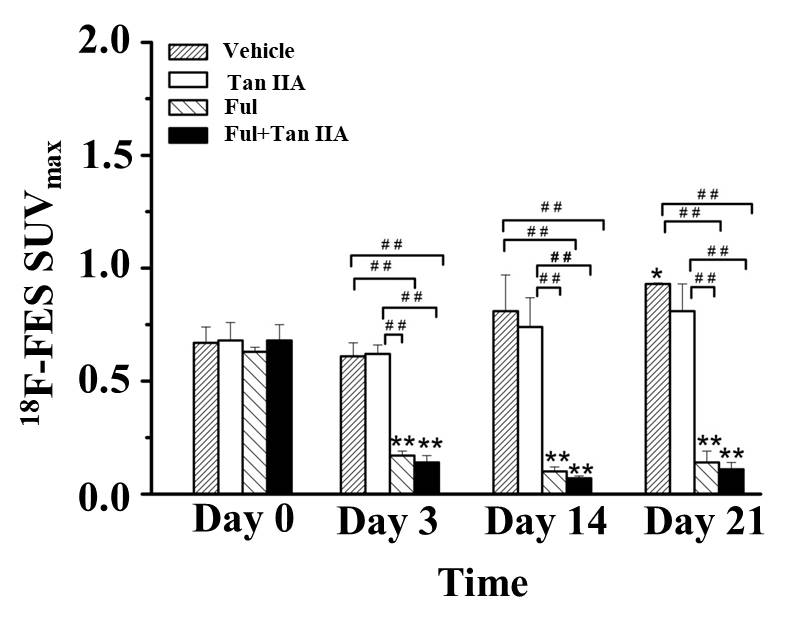


Fig S1. Quantitative analysis of ^18^F-FES uptake SUV from the microPET/CT imaging on days 0, 3, 14, and 21 after treatment. * *P* < 0.05, ** *P* < 0.001, within groups compared to baseline; # *P* < 0.05, ## *P* < 0.001 between treatment and vehicle groups.
